# GLOBE: an explainable machine learning platform for preoperative prediction of thromboembolism and neurological deterioration in patients with glioma

**DOI:** 10.3389/fnins.2026.1801112

**Published:** 2026-06-01

**Authors:** Feiling Xiang, Xuelian Yang, Sijin Xiang, Mengyuan Fu, Gang Yang

**Affiliations:** 1 Department of Neurosurgery, The First Affiliated Hospital of Chongqing Medical University, Chongqing, China; 2 Department of Nursing, The First Affiliated Hospital of Chongqing Medical University, Chongqing, China

**Keywords:** glioma, machine learning, postoperative neurological deterioration, SHAP, venous thromboembolism

## Abstract

**Background:**

Patients with glioma are at high risk of postoperative venous thromboembolism (VTE) and postoperative neurological deterioration (PND). Conventional clinical scoring systems have limited accuracy in predicting these perioperative risks. This study aimed to develop and validate machine-learning models for individualized preoperative prediction of postoperative VTE and PND in patients with glioma.

**Methods:**

A retrospective cohort of 427 patients with glioma was included. Patients were randomly divided into training and test sets at an 8:2 ratio using stratified random sampling. Multiple machine-learning algorithms were trained and evaluated. Model performance was assessed using the area under the curve (AUC), accuracy, sensitivity, specificity, calibration curves, and decision curve analysis. An online prediction platform was developed to facilitate individualized risk assessment.

**Results:**

Among 427 patients, postoperative VTE and PND occurred in 34 and 35%, respectively. For VTE prediction, the final Top-10 random forest model outperformed the Caprini score alone and achieved an AUC of 0.815 (95% CI, 0.720–0.910) in the held-out test set. Performance remained strong in the clinically significant VTE sensitivity analysis (AUC, 0.923; 95% CI, 0.847–0.998). SHAP analysis indicated that older age, elevated D-dimer and fibrin degradation products (FDP), as well as lower hemoglobin levels, were associated with increased predicted VTE risk. For PND prediction, the final Top-10 logistic regression model achieved an AUC of 0.741 (95% CI, 0.627–0.854). Older age, recurrent glioma, higher Caprini score, higher neutrophil percentage, and hypertension history tended to increase predicted PND risk. Models were deployed in the GLOBE web platform (https://gliomas.shinyapps.io/GLOBE/) for real-time preoperative risk prediction.

**Conclusion:**

We developed accurate, interpretable, and clinically meaningful preoperative prediction models for postoperative VTE and PND in patients with glioma. The GLOBE online prediction system translates these models into a practical tool for individualized perioperative risk stratification.

## 1 Introduction

Glioma is one of the most common primary malignant tumors of the central nervous system, with an estimated incidence of approximately 5.5 per 100,000 person-years (Ryan et al., 2020). Despite advances in diagnostic technologies, surgical techniques, radiotherapy, and chemotherapy, glioma remains associated with substantial morbidity and mortality (Louis et al., 2021; Ostrom et al., 2023). For example, most malignant gliomas are associated with an average survival of approximately 14 months after diagnosis (Van Meir et al., 2010). Moreover, patient outcomes vary widely even with standard resection and adjuvant therapy because tumor biology and patient condition are highly heterogeneous. In particular, perioperative complications represent an important and potentially modifiable contributor to this variability (Khorana et al., 2007; Rahman et al., 2016). Therefore, improved risk stratification and early identification of high-risk patients of perioperative complications are essential.

Venous thromboembolism (VTE), including deep vein thrombosis (DVT) and pulmonary embolism (PE), is a frequent and potentially fatal complication in patients with glioma (Semrad et al., 2007). Hypercoagulability, surgical trauma, prolonged immobilization, and systemic inflammation contribute to the elevated thrombotic risk (Ay et al., 2017; Streiff et al., 2021). Previous studies have reported postoperative VTE incidence rates ranging from 3% to over 30% in glioma patients (Marras et al., 2000; Simanek et al., 2007). Importantly, VTE has been shown not only to increase perioperative morbidity but also to adversely affect long-term survival (Chew et al., 2006). Although risk assessment tools such as the Caprini score are widely used in surgical populations, their predictive accuracy and clinical utility in neurosurgical oncology remain limited (Pannucci et al., 2017). For example, a previous retrospective observational cohort study showed that the Caprini score has low positive predictive value and is difficult to interpret in neurosurgical practice (Maria et al., 2024). These limitations likely arise because the Caprini score relies on manually selected variables with fixed weights. As a result, it does not capture non-linear relationships or complex interactions between predictors. This underscores the need for more flexible, data-driven approaches for individualized risk prediction in oncological populations.

Postoperative neurological deterioration (PND) represents another major concern following glioma surgery. PND, typically defined as new or worsened neurological deterioration after surgery, is associated with reduced functional independence, delayed adjuvant therapy, and impaired quality of life (Duffau, 2006; McGirt et al., 2009). Despite its clinical importance, reliable perioperative prediction of PND remains challenging, and existing models are often constrained by limited feature integration and modest predictive performance.

Against this background, the present study aimed to develop and validate machine learning (ML) based prediction models for two clinically critical perioperative outcomes in patients undergoing glioma surgery: VTE and PND. We systematically compared multiple ML algorithms, assessed model discrimination, calibration, and clinical utility. The finalized models were translated into an online prediction system, termed GLOBE (GLiOma platform for individualized prediction of postoperative Binary Events), to provide real-time, individualized risk assessment. In clinical practice, accurate preoperative prediction of VTE risk may support risk-adapted thromboprophylaxis strategies and closer postoperative surveillance. Similarly, predicting PND risk may facilitate individualized perioperative management and early rehabilitation planning.

## 2 Materials and methods

### 2.1 Study design and population

This retrospective study enrolled adult patients diagnosed with glioma who underwent surgical resection at The First Affiliated Hospital of Chongqing Medical University between July 1, 2022, and September 30, 2025. Patients were eligible for inclusion if complete preoperative clinical data and postoperative outcome information were available. The study was conducted in accordance with the Declaration of Helsinki and was approved by the Institutional Review Board of The First Affiliated Hospital of Chongqing Medical University (No. 2025-667-01). Informed consent was waived due to the study’s retrospective nature.

Patients were eligible for inclusion if they met all of the following criteria: (1) age ≥ 18 years; (2) pathologically or clinically diagnosed glioma; (3) underwent glioma resection; and (4) available preoperative clinical and laboratory variables and postoperative outcome data (VTE and PND). Patients were excluded if they met any of the following criteria: (1) no surgical treatment; (2) missing preoperative variables or postoperative outcomes; or (3) duplicated records or repeated admissions. A total of 457 adult patients with glioma underwent surgical resection. After excluding 30 with missing postoperative outcome data, 427 remained in the final cohort. No missing values were observed in the preoperative variables of included patients, and no imputation was therefore required.

### 2.2 Data collection and variables

The primary outcomes of interest were (1) postoperative VTE, defined as objectively confirmed DVT and/or PE occurring within 30 days after surgery, and (2) PND, defined as new-onset or worsening impairment in the level of consciousness, aphasia, or motor function, as assessed by standardized neurological examination within 30 days after surgery. Routine postoperative ultrasonographic screening of lower-extremity veins was performed before and after surgery.

To develop prediction models for VTE and PND, only variables available before surgery were included. All intraoperative and postoperative variables were excluded to ensure temporal validity and to avoid post-treatment information leakage. Preoperative data were extracted from electronic medical records. Candidate predictors comprised: demographic and lifestyle characteristics (*n* = 5), including age, sex, body mass index, smoking history, and alcohol history; medical comorbidities and thrombotic history (*n* = 6), including hypertension, diabetes, hyperlipidemia, coronary heart disease, stroke, and prior VTE; preoperative risk assessment variables (*n* = 2), including preoperative anticoagulant use and the preoperative Caprini score; tumor-related features (*n* = 5), including tumor location, laterality, extent of spread, maximum diameter, and recurrence status; and a comprehensive panel of preoperative laboratory tests (*n* = 30), covering coagulation parameters, hematological indices, inflammatory markers, liver and renal function tests, electrolyte levels, and metabolic profiles. Detailed definitions of all variables are provided in Supplementary Tables 1, 2.

### 2.3 Model development for VTE and PND

Separate prediction models were developed for postoperative VTE and PND, both treated as binary outcomes. The final analytic cohort was divided into a training set (80%) and an independent test set (20%) using a fixed stratified random split. The test set was held out from all model development procedures and used exclusively for final evaluation.

For each outcome, candidate predictors were initially screened using univariate logistic regression analyses conducted within the training set only. Because this step was intended for preliminary dimensionality reduction rather than formal etiologic inference, unadjusted *p* < 0.05 was used as the preselection criterion, consistent with common practice in machine-learning–based prediction pipelines (Chowdhury and Turin, 2020). A random forest model was then fitted to rank variable importance, and reduced feature sets were constructed based on predictor rankings. For VTE prediction, models were developed using Top-5, Top-10, and Top-13 feature sets, with the preoperative Caprini score included as a comparator. For PND prediction, models were developed using Top-5 and Top-10 feature sets.

Four classification algorithms were evaluated for each feature set: generalized linear model (GLM), random forest (RDF), gradient boosting algorithm (GBA), and support vector machine with a radial basis function kernel (SVM). Model training and hyperparameter tuning were conducted in the training set using repeated 5-fold cross-validation (5-folds, 5 repeats), with AUC as the primary optimization metric. After model fitting, performance was assessed on the full held-out test set. Evaluation metrics included AUC with 95% confidence interval, accuracy, sensitivity, and specificity. On the basis of overall discrimination, parsimony, and downstream validation results, the Top-10 RDF model was selected as the final model for VTE prediction, and the Top-10 GLM model was selected as the final model for PND prediction.

### 2.4 Calibration, clinical utility, and model interpretation

For the final selected models, calibration was evaluated in the held-out test set using calibration plots and the Brier score. Clinical utility was assessed by decision curve analysis (DCA), in which model-derived net benefit was examined across a range of threshold probabilities and compared with default strategies (treat-all and treat-none). For VTE, the final machine-learning model was additionally compared with the preoperative Caprini score.

Model interpretability was assessed using Shapley Additive Explanations (SHAP). SHAP analyses were performed on the final selected models to quantify each predictor’s relative contribution and visualize the magnitude and direction of feature effects on individualized risk predictions.

Because the institutional VTE surveillance protocol may detect distal or asymptomatic thrombotic events, a sensitivity analysis was performed by restricting the endpoint to clinically significant VTE to further assess the robustness and clinical relevance of the final model. Clinically significant VTE was operationally defined as pulmonary embolism or thrombosis involving named deep veins (e.g., posterior tibial or fibular/peroneal veins), which are more likely to be symptomatic or clinically actionable. Isolated distal lower-extremity thrombotic events, such as intramuscular calf vein thrombosis, were classified as non-clinically significant.

### 2.5 Model implementation and web deployment

The final selected models were implemented in an interactive web-based application using Shiny, termed GLOBE, accessible at https://gliomas.shinyapps.io/GLOBE/. The platform incorporates the Top-10 random forest model for VTE prediction and the Top-10 generalized linear model for PND prediction. Users can enter patient-specific preoperative clinical and laboratory variables to obtain real-time predicted probabilities for both outcomes. The web application is intended as a decision-support tool for perioperative risk stratification rather than a stand-alone treatment decision system.

## 3 Results

### 3.1 Patient characteristics

**FIGURE 1:**
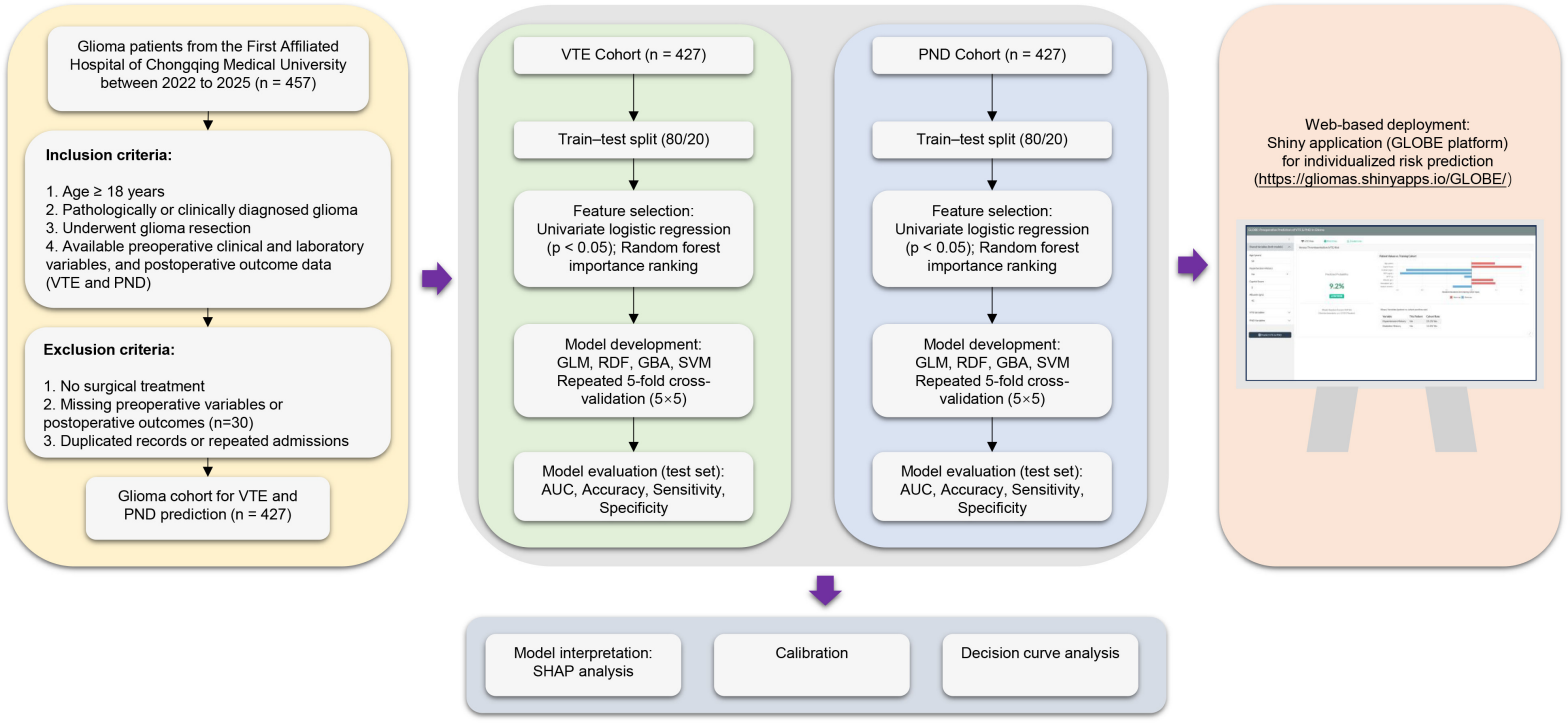
Study workflow. Flowchart showing patient selection, data splitting (80:20), feature selection, model development, evaluation on the held-out test set, SHAP-based interpretation, and deployment via the GLOBE platform.

The workflow of the study is illustrated in Figure 1. For VTE prediction, a total of 427 patients were included in the final analysis after applying the inclusion and exclusion criteria. Among them, 342 patients were allocated to the training set and 85 to the testing set using an 8:2 split. The mean age in the training cohort was 53 ± 14 years, and the cohort comprised 180 male and 162 female patients with glioma. Postoperative VTE occurred in 34% of patients. The six most common VTE locations were bilateral calf intramuscular vein thrombosis, left calf intramuscular vein thrombosis, left posterior tibial vein thrombosis, pulmonary embolism, right calf intramuscular vein thrombosis, and right peroneal vein thrombosis. For patients with multiple VTE events, only the time from surgery to the first event was recorded. The median time to any VTE occurrence was 5 days postoperatively, with the longest recorded interval being 24 days. Of all VTE events, 40% were detected within 3 days of surgery, which is attributable to the institutional protocol of routine postoperative lower-extremity ultrasonographic screening that systematically identifies both symptomatic and asymptomatic thrombotic events at an early postoperative timepoint. Among all 427 patients, clinically significant VTE (defined as pulmonary embolism, posterior tibial vein thrombosis, or fibular/peroneal vein thrombosis) occurred in 13% of the cohort, with a median time to onset of 5 days after surgery. Baseline demographic characteristics, tumor-related features, perioperative variables, and laboratory tests are summarized in Supplementary Table 1. For PND prediction, 35% of patients experienced PND (121 of 342 in the training set and 30 of 85 in the test set). The mean age in the training set was 53 ± 14 years, comprising 177 male and 165 female patients. Baseline characteristics are summarized in Supplementary Table 2.

**FIGURE 2:**
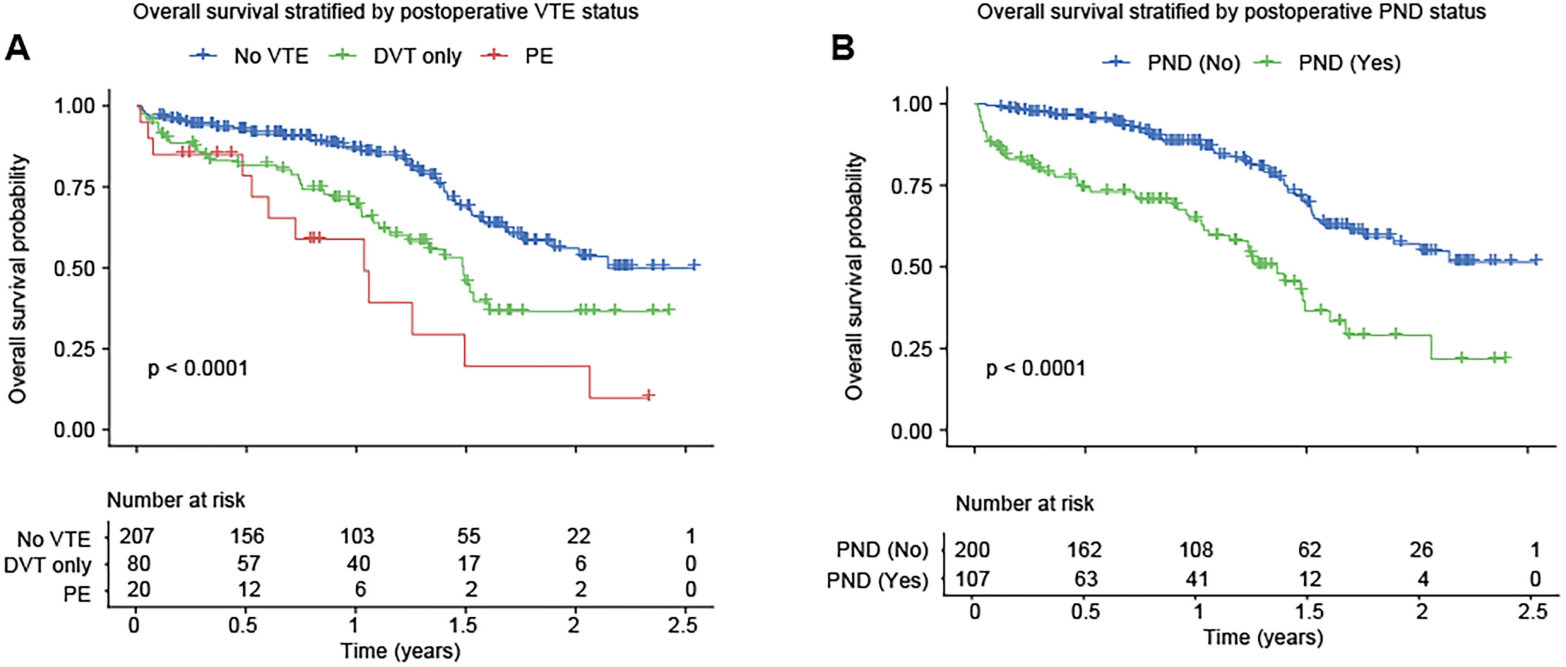
Postoperative outcomes and their association with overall survival. **(A)** Overall survival stratified by postoperative VTE status (no VTE, DVT only, and PE). **(B)** Overall survival stratified by PND status. *P*-values were calculated using the log-rank test. Abbreviations: VTE, venous thromboembolism; DVT, deep vein thrombosis; PE, pulmonary embolism; PND, postoperative neurological deterioration.

To show the clinical importance of preventing VTE and PND, we examined their association with overall survival. Of the 427 patients, 307 had available overall survival data and were included in the Kaplan–Meier analysis. Kaplan–Meier analysis demonstrated significant differences in OS according to postoperative VTE status (Figure 2A). Patients without VTE had the most favorable survival, whereas those with DVT alone showed intermediate outcomes, and those with PE had the poorest prognosis. Similarly, postoperative PND was associated with significantly reduced OS compared with patients without PND (Figure 2B).

### 3.2 Performance of VTE prediction models

#### 3.2.1 Discrimination and classification performance

**FIGURE 3:**
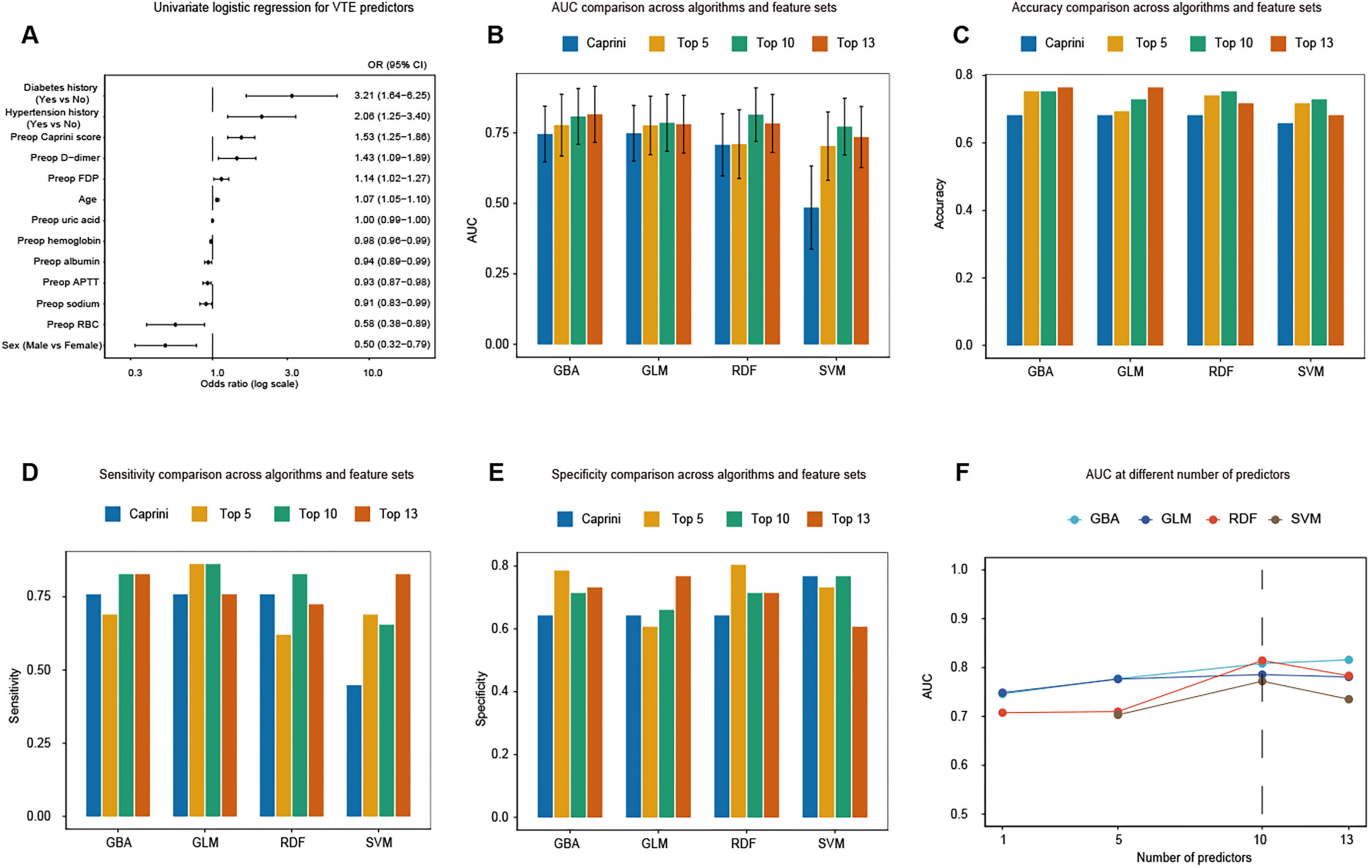
Feature selection and predictive performance of machine-learning models for postoperative VTE prediction. **(A)** Univariate logistic regression for VTE predictors. Forest plot showing odds ratios (ORs) and 95% confidence intervals (CIs) for preoperative variables significantly associated with postoperative VTE. Variables with OR > 1 indicate increased risk, and those with OR < 1 indicate decreased risk. **(B)** AUC comparison across algorithms and feature sets in the held-out test set. **(C)** Accuracy comparison across algorithms and feature sets in the held-out test set. **(D)** Sensitivity comparison across algorithms and feature sets in the held-out test set. **(E)** Specificity comparison across algorithms and feature sets in the held-out test set. **(F)** AUC values for different numbers of predictors. Line plot showing how AUC varied across algorithms as the number of selected predictors increased from 1 (Caprini score alone) to 13. Abbreviations: AUC, area under the curve; GLM, generalized linear model; RDF, random forest; GBA, gradient boosting algorithm; SVM, support vector machine.

Univariate logistic regression analysis identified 13 preoperative variables significantly associated with postoperative VTE (Figure 3A). Older age (OR = 1.07, 95% CI: 1.05–1.10), a higher preoperative Caprini score (OR = 1.53, 95% CI: 1.25–1.86), diabetes history (OR = 3.21, 95% CI: 1.64–6.25), and hypertension history (OR = 2.06, 95% CI: 1.25–3.40) were associated with increased odds of postoperative VTE. In contrast, higher preoperative hemoglobin, activated partial thromboplastin time (APTT), red blood cell (RBC) count, albumin, and sodium were associated with lower VTE risk. Elevated preoperative D-dimer, fibrin degradation products (FDP), and uric acid were also associated with increased risk.

Across algorithms and feature sets, machine-learning models using Top-10 or Top-13 variables showed better discrimination than models using Top-5 variables or the Caprini score alone (Figures 3B–E) in the test dataset. The highest AUC in the independent test set was achieved by the Top-13 GBA model (AUC 0.816, 95% CI 0.716–0.915), closely followed by the Top-10 RDF model (AUC 0.815, 95% CI 0.720–0.910). Because the Top-10 RDF model achieved near-maximal discrimination with fewer variables and was selected for downstream interpretation and deployment, it was retained as the final VTE model (Figure 3F). Using the Youden-optimal threshold (0.313), the final Top-10 RDF model achieved an accuracy of 0.753, sensitivity of 0.828, and specificity of 0.714 in the test set.

#### 3.2.2 Calibration, clinical utility, and SHAP interpretation of the final VTE model

**FIGURE 4:**
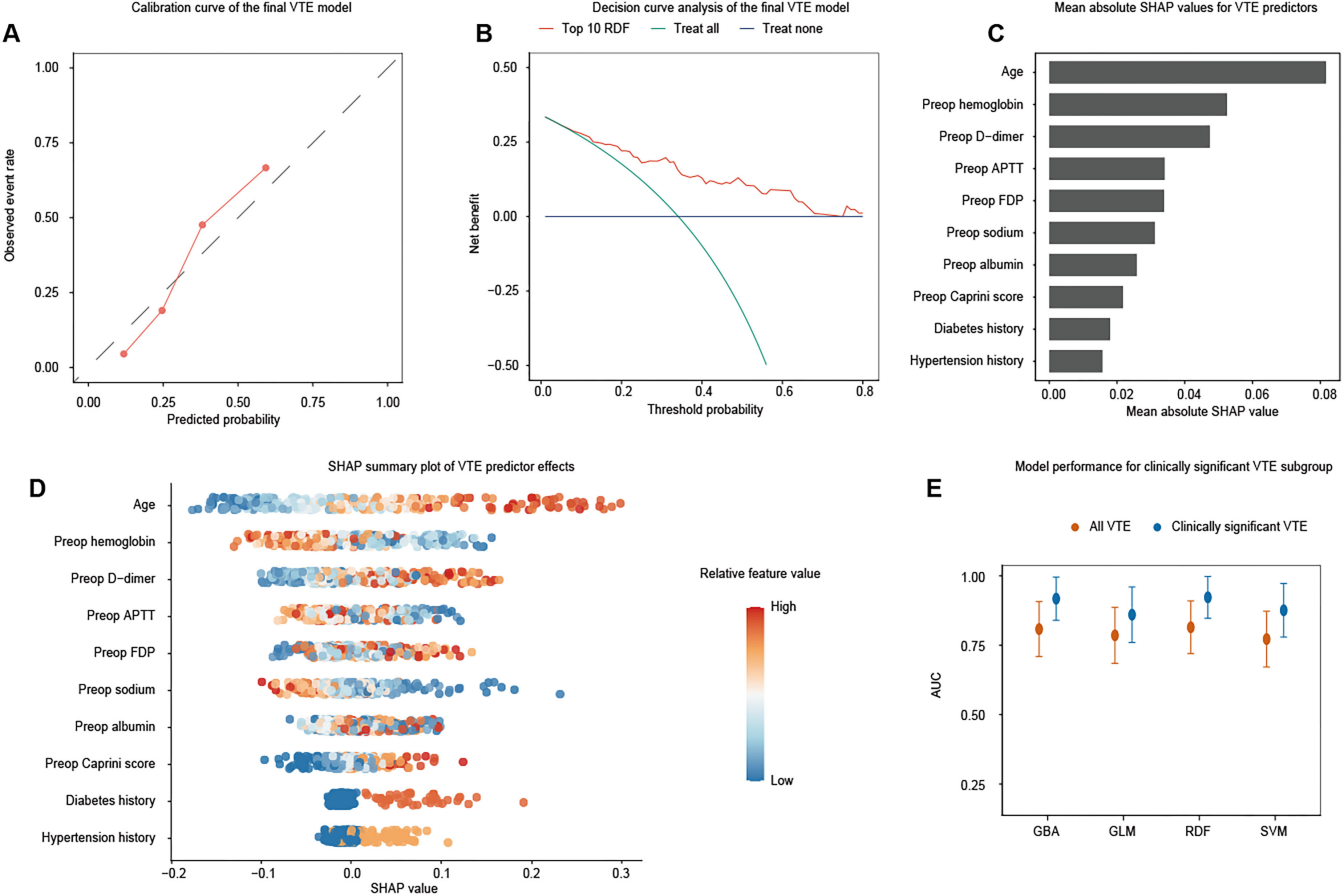
Calibration, clinical utility, SHAP-based interpretation, and sensitivity analysis of the final VTE model. **(A)** Calibration curve of the final VTE model. Calibration plot of the Top-10 RDF model in the held-out test set. The plot shows the agreement between predicted probabilities and observed event rates. **(B)** Decision curve analysis of the final VTE model. Net benefit of the Top-10 RDF model across a range of threshold probabilities, compared with the treat-all and treat-none strategies. **(C)** Mean absolute SHAP values for VTE predictors. Bar chart showing the mean absolute SHAP value for each predictor in the final model. The values reflect the relative contribution of each variable to model predictions. **(D)** SHAP summary plot of VTE predictor effects. Beeswarm plot showing the distribution and direction of SHAP values for each predictor across all patients. Each point represents one patient. Color indicates the relative feature value (red = high, blue = low). For categorical variables, the color scale reflects encoded category levels rather than continuous magnitudes. For binary variables, red (“high”) indicates the presence of the characteristic (e.g., history = yes, coded as 1), whereas blue (“low”) indicates its absence (coded as 0). **(E)** Model performance for clinically significant VTE subgroup. Comparison of AUC across all four algorithms for the sensitivity analysis, restricted to the clinically significant VTE as the endpoint. Abbreviations: SHAP, Shapley Additive Explanations; FDP, fibrin degradation products; APTT, activated partial thromboplastin time.

The calibration plot for the Top-10 RDF model showed good agreement between predicted probabilities and observed event rates in the test set, with a Brier score of 0.165 (Figure 4A). Decision curve analysis demonstrated that the Top-10 RDF model provided greater net benefit than the treat-all and treat-none strategies across a broad range of threshold probabilities, with positive incremental net benefit observed approximately from 0.06 to 0.80 (Figure 4B).

SHAP analysis further improved the interpretability of the final model. The most influential features were age, preoperative hemoglobin, preoperative D-dimer, preoperative APTT, preoperative FDP, preoperative sodium, preoperative albumin, preoperative Caprini score, diabetes history, and hypertension history (Figure 4C). The SHAP summary plot showed that older age, higher preoperative D-dimer, higher FDP levels, and preoperative Caprini score, diabetes history, and hypertension history were generally associated with higher predicted VTE risk. In contrast, higher preoperative hemoglobin, sodium, and APTT were generally associated with lower predicted VTE risk (Figure 4D).

To further assess clinical relevance, we performed a sensitivity analysis restricting the VTE endpoint to symptomatic events only. In the held-out test set of 85 patients, 11 had clinically significant VTE and 74 did not. Among the 29 patients with any VTE in the test set, 11 were clinically significant, and 18 were non–clinically significant. All four Top-10 algorithms demonstrated strong discrimination for clinically significant VTE: GBA achieved an AUC of 0.918 (95% CI 0.840–0.995), GLM 0.860 (95% CI 0.760–0.960), RDF 0.923 (95% CI 0.847–0.998), and SVM 0.876 (95% CI 0.779–0.972) (Figure 4E).

### 3.3 Performance of PND prediction models

#### 3.3.1 Feature selection and discrimination performance

**FIGURE 5:**
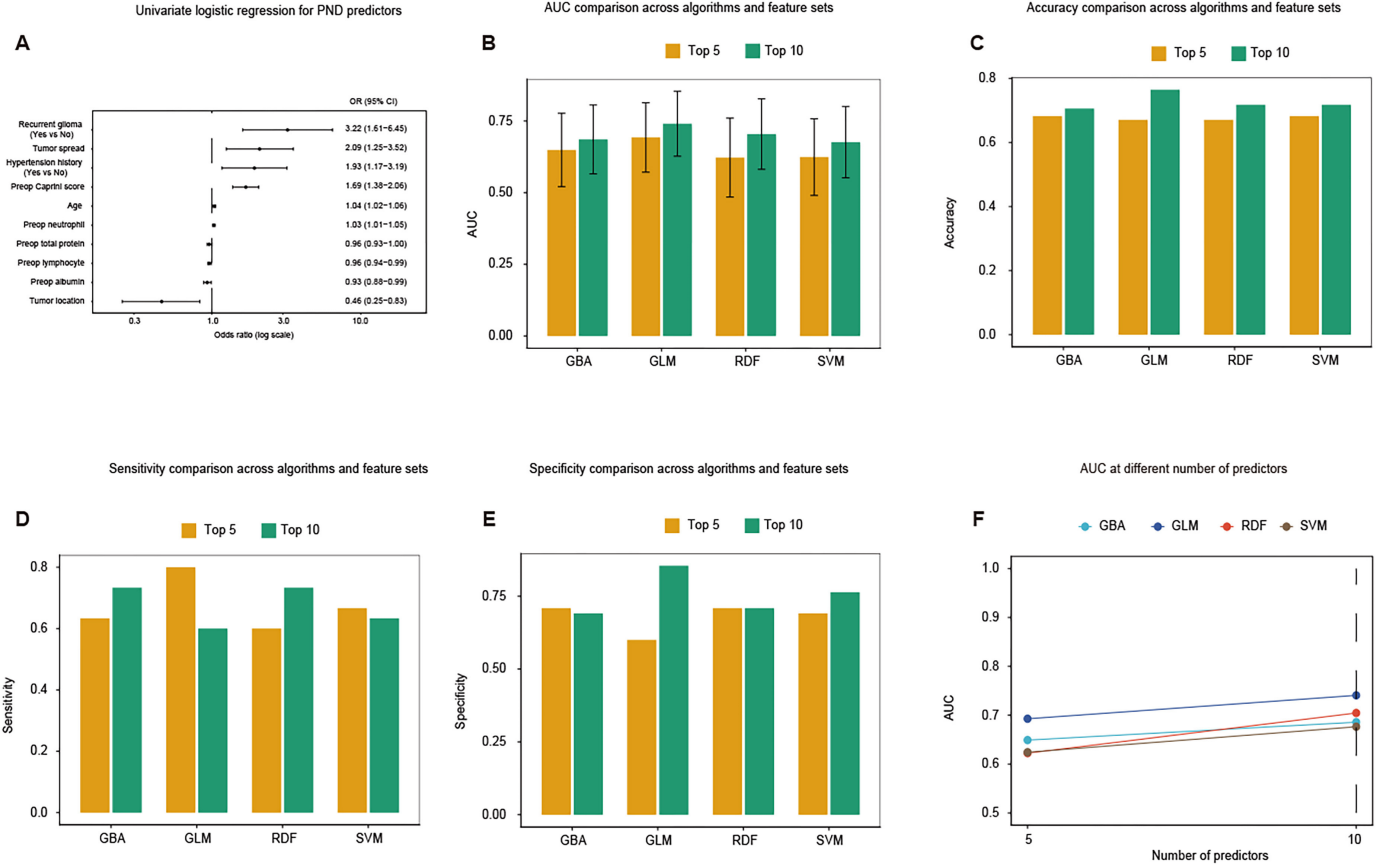
Feature selection and predictive performance of machine-learning models for postoperative neurological deterioration prediction. **(A)** Univariate logistic regression for PND predictors. Forest plot showing odds ratios (ORs) and 95% confidence intervals (CIs) for preoperative variables significantly associated with postoperative neurological deterioration (PND). **(B)** AUC comparison across algorithms and feature sets in the held-out test set. **(C)** Accuracy comparison across algorithms and feature sets in the held-out test set. **(D)** Sensitivity comparison across algorithms and feature sets in the held-out test set. **(E)** Specificity comparison across algorithms and feature sets in the held-out test set. **(F)** AUC values for different numbers of predictors. Line plot showing how AUC varied across algorithms as the number of selected predictors increased from 5 to 10.

Univariate logistic regression analysis identified 10 preoperative variables significantly associated with postoperative PND (Figure 5A). A higher preoperative Caprini score (OR = 1.69, 95% CI: 1.38–2.06), older age (OR = 1.04, 95% CI: 1.02–1.06), recurrent glioma (OR = 3.22, 95% CI: 1.61–6.45), regional tumor spread (OR = 2.09, 95% CI: 1.25–3.52), higher neutrophil percentage, and hypertension history were associated with increased odds of PND. In contrast, higher lymphocyte percentage, higher albumin, and higher total protein were associated with lower risk.

Models using the Top-10 feature set consistently outperformed the corresponding Top-5 models in the independent test set (Figures 5B–E). Among all candidate PND models, the Top-10 GLM achieved the best overall discrimination, with an AUC of 0.741 (95% CI 0.627–0.854) (Figure 5F). At the Youden-optimal threshold of 0.505, this model achieved an accuracy of 0.765, sensitivity of 0.600, and specificity of 0.855. Therefore, the Top-10 GLM was selected as the final PND model.

#### 3.3.2 Calibration, clinical utility, and SHAP interpretation of the final PND model

**FIGURE 6:**
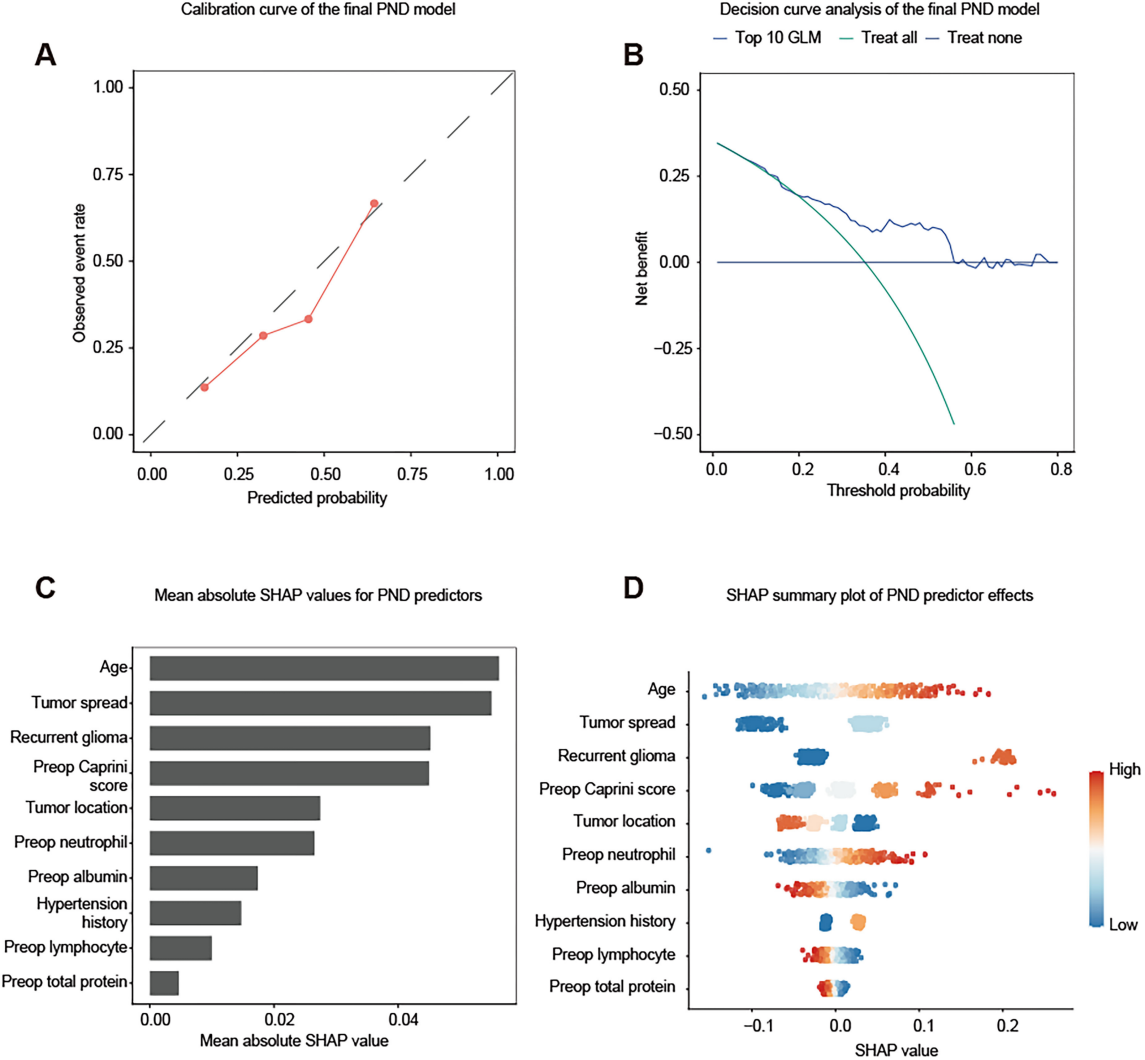
Calibration, clinical utility, and SHAP-based interpretation of the final PND model. **(A)** Calibration curve of the final PND model. Calibration plot of the Top-10 GLM model in the held-out test set. It demonstrates the agreement between predicted probabilities and observed event rates. **(B)** Decision curve analysis of the final PND model. Net benefit of the Top-10 GLM model across a range of threshold probabilities, compared with the treat-all and treat-none strategies. **(C)** Mean absolute SHAP values for PND predictors. Bar chart showing the mean absolute SHAP value for each predictor in the final model. **(D)** SHAP summary plot of PND predictor effects. Beeswarm plot showing the distribution and direction of SHAP values for each predictor across all patients. Each point represents one patient. Color indicates the relative feature value (red = high, blue = low). For categorical variables, the color scale reflects encoded category levels rather than continuous magnitudes. For binary variables, red (“high”) indicates the presence of the characteristic (e.g., history = yes, coded as 1), whereas blue (“low”) indicates its absence (coded as 0).

The calibration curve of the final Top-10 GLM model showed acceptable agreement between predicted and observed risks in the held-out test set, with a Brier score of 0.195 (Figure 6A). Decision curve analysis showed that the Top-10 GLM model provided higher net benefit than the treat-all and treat-none strategies across a wide range of threshold probabilities, with a favorable net benefit of approximately from 0.08 to 0.77 (Figure 6B).

SHAP-based interpretation showed that age, tumor spread, recurrent glioma, preoperative Caprini score, tumor location, neutrophil percentage, preoperative albumin, hypertension history, lymphocyte percentage, and total protein were the most important contributors to model predictions (Figure 6C). In the SHAP summary plot, older age, regional tumor spread, recurrent glioma, higher Caprini score, higher neutrophil percentage, and hypertension history tended to increase predicted PND risk. In contrast, higher albumin, lymphocyte percentage, total protein, and favorable tumor location were associated with lower predicted risk (Figure 6D).

### 3.4 Web-based implementation

**FIGURE 7:**
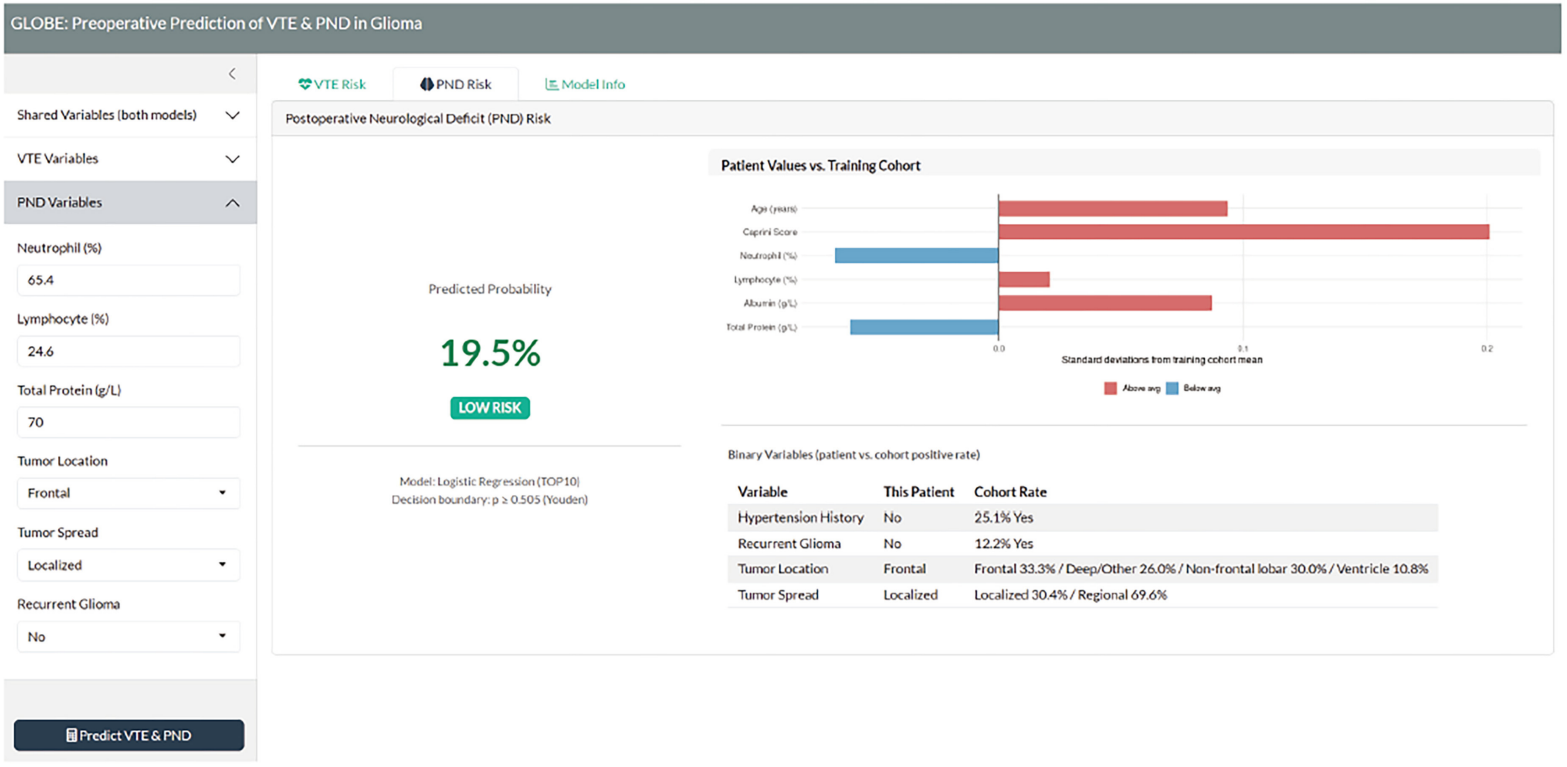
The GLOBE web-based prediction platform for individualized risk assessment. Representative screenshot of the GLOBE (GLiOma platform for individualized prediction of postoperative Binary Events) web application, accessible at https://gliomas.shinyapps.io/GLOBE/. The platform incorporates the Top-10 random forest model for VTE prediction and the Top-10 generalized linear model for PND prediction. Users enter patient-specific preoperative clinical and laboratory variables in the left panel and obtain real-time, individualized predicted probabilities for both postoperative VTE and PND, along with a comparison of the patient values relative to the training cohort distribution.

The final VTE and PND models were deployed in an interactive Shiny-based web platform, GLOBE,^1^ to support individualized preoperative risk estimation in patients with glioma (Figure 7). Specifically, the platform incorporates the Top-10 random forest model for VTE prediction and the Top-10 generalized linear model for PND prediction. By entering patient-specific preoperative clinical and laboratory variables, users can obtain real-time predicted probabilities for both postoperative outcomes.

## 4 Discussion

In this study, we developed and validated two preoperative prediction models for clinically important postoperative complications in patients with glioma: venous thromboembolism (VTE) and postoperative neurological deterioration (PND). Using only preoperative clinical and laboratory variables only, the final Top-10 random forest model (RDF) for VTE and the final Top-10 logistic regression model (GLM) for PND demonstrated favorable discrimination, calibration, clinical utility, and interpretability in the held-out test set. These models were further deployed through the GLOBE web platform to facilitate individualized perioperative risk stratification.

Prior neurosurgical studies have reported a particularly high thrombotic risk among patients with glioma (Perry et al., 2010). In our study, a relatively high VTE incidence of 34% was observed, which is primarily attributable to the routine postoperative ultrasonographic screening protocol employed at our local institution, which systematically identified distal intramuscular vein thrombosis in addition to more proximal events. Distal DVT is generally considered less clinically significant than proximal DVT or PE and may be managed conservatively in many settings (Galanaud et al., 2009; Kirkilesis et al., 2020). However, accumulating evidence suggests that isolated distal DVT is not entirely benign and may progress to proximal thrombosis or PE, particularly in high-risk populations such as cancer patients (Galanaud et al., 2024). The optimal management of distal DVT remains debated internationally, and regional practice varies considerably. In certain Chinese centers, including our institution, anticoagulation is recommended even for isolated distal or intramuscular vein thrombosis (Yicheng and Changming, 2021), which reflects a more proactive thromboprophylaxis strategy in high-risk neurosurgical patients. For patients identified as high-risk by the GLOBE platform, closer postoperative surveillance, early mechanical prophylaxis, and individualized consideration of pharmacological anticoagulation may be warranted.

A previous study constructed a nomogram for predicting acute VTE after glioma surgery and achieved an AUC of 0.788 (95% CI, 0.69–0.89) in the validation set (Zhang et al., 2023). Compared with prior prediction models, the present study offers several methodological and practical advances. First, by employing machine learning algorithms with repeated cross-validation and systematic feature selection, the final Top-10 random forest model achieved a comparable or superior AUC of 0.815 (95% CI, 0.720–0.910) in the held-out test set, and performed even more strongly when restricted to clinically significant VTE (AUC 0.923; 95% CI, 0.847–0.998). Second, a major methodological strength of the present study is the strict restriction of all predictors to variables available before surgery. By explicitly excluding all intraoperative and postoperative variables, we improved temporal validity and eliminated the risk of information leakage. This ensures that our model is fully aligned with its intended clinical use as a preoperative risk stratification tool. Third, to maximize clinical accessibility, the final models were deployed in the GLOBE web platform (see text footnote 1), which allows clinicians to obtain individualized predicted probabilities for both VTE and PND in real time from any device, without requiring statistical software or manual nomogram calculation.

The key VTE-associated predictors identified in our study align well with the existing literature. Elevated preoperative D-dimer and FDP reflect an underlying hypercoagulable state and have been independently associated with postoperative VTE risk in multiple surgical oncology cohorts (Li et al., 2019). This published study also showed an AUC value of 0.628 when D-dimer was used alone, which increased to 0.777 with the addition of platelet distribution width (PDW). Lower preoperative hemoglobin and albumin levels, reflecting hematological vulnerability and nutritional depletion, have similarly been linked to increased thrombotic risk in both surgical and cancer populations (Valeriani et al., 2024). Furthermore, diabetes history and hypertension have been recognized as systemic comorbidities that promote a prothrombotic milieu and are important risk factors of postoperative VTE (Huang et al., 2016).

PND occurred in over one-third of patients and was strongly associated with reduced OS. Previous studies have shown that early postoperative neurological decline leads to prolonged hospitalization, delayed adjuvant therapy, compromised functional recovery, and a decrease in overall survival (Rahman et al., 2016). However, few studies have attempted to predict PND using preoperative data alone. For PND prediction, our model emphasized age, tumor spread, recurrent glioma, preoperative Caprini score, tumor location, neutrophil percentage, albumin, hypertension history, lymphocyte percentage, and total protein. These variables likely reflect both disease complexity and patient vulnerability. Tumor spread and recurrent disease may indicate greater anatomical complexity and surgical difficulty, whereas albumin, total protein, and leukocyte-related indices may capture nutritional and inflammatory status that could influence perioperative neurological recovery and survival (Huq et al., 2021). These findings are broadly consistent with prior literature documenting that functional vulnerability, systemic inflammation, and tumor invasiveness are key determinants of postoperative neurological outcomes in glioma patients.

The GLOBE web platform is intended as a practical decision-support interface for individualized risk stratification rather than a stand-alone treatment decision system. For patients identified as high VTE risk (> 0.313) by the GLOBE platform, closer postoperative surveillance, early mechanical prophylaxis (including compression stockings and intermittent pneumatic compression), early ambulation, and individualized consideration of pharmacological anticoagulation with low-molecular-weight heparin may be warranted, in accordance with current neurosurgical thromboprophylaxis guidelines. A high predicted PND risk (0.505) may inform preoperative counseling, perioperative monitoring, intraoperative strategy, and early rehabilitation planning. Model outputs should always be interpreted in conjunction with clinical judgment and institutional context. By enabling real-time individualized predictions, GLOBE may assist clinicians in optimizing thromboprophylaxis strategies, surgical planning, and perioperative risk counseling.

This study has several limitations. First, as a retrospective single-center analysis, its findings require validation in multicenter prospective cohorts before broader clinical implementation can be considered. Second, the definition of the VTE endpoint was based on our institutional surveillance framework and may include events that differ from the narrower and more clinically consequential definitions used in other settings. Although the sensitivity analysis was restricted to clinically significant VTE, heterogeneity in endpoint definitions across institutions remains an important concern. Third, key molecular markers, including IDH mutation status, MGMT promoter methylation, and TERT mutation, were not incorporated into the analysis. Fourth, the relatively low event-per-variable ratio in the PND model, despite partial mitigation through cross-validation and machine-learning-based feature selection, may still introduce a residual risk of overfitting. Future studies involving larger and more diverse cohorts are warranted to further establish the robustness and generalizability of these models.

## 5 Conclusion

In conclusion, we developed two interpretable preoperative prediction models for postoperative VTE and PND in patients with glioma using routinely available clinical and laboratory variables. The final random forest model for VTE and logistic regression model for PND showed favorable performance and were implemented in the GLOBE web platform. These findings support the potential value of individualized perioperative risk stratification, although external validation is required before wider clinical adoption.

## Data Availability

The datasets and code used for analysis in this study are available in the GitHub repository at: https://github.com/NeuroCQ/VTE. Further inquiries can be directed to the corresponding author.

## Footnotes

1
https://gliomas.shinyapps.io/GLOBE/
