# Supplementary material for: GLOBE: an explainable machine learning platform for preoperative prediction of thromboembolism and neurological deterioration in patients with glioma
Source: Front Neurosci. 2026 Jun 1;20:1801112. doi: 10.3389/fnins.2026.1801112 (PMC13265528; doi:10.3389/fnins.2026.1801112)
Supplement: Supplementary file 1 [file Table_1.docx]

| **Characteristic** | **Train**  N = 342^1^ | **Test**  N = 85^1^ | **p-value**^2^ |
| --- | --- | --- | --- |
| **VTE** |  |  | >0.9 |
| No | 226 (66%) | 56 (66%) |  |
| Yes | 116 (34%) | 29 (34%) |  |
| **Clinically significant VTE** |  |  | >0.9 |
| No | 297 (87%) | 74 (87%) |  |
| Yes | 45 (13%) | 11 (13%) |  |
| **Age (per year)** | 53 ± 14 | 51 ± 16 | 0.2 |
| **Sex** |  |  | 0.3 |
| Female | 162 (47%) | 46 (54%) |  |
| Male | 180 (53%) | 39 (46%) |  |
| **BMI (kg/m^2)** | 23.53 ± 2.82 | 23.38 ± 2.96 | 0.4 |
| **Smoking history** |  |  | 0.8 |
| No | 269 (79%) | 66 (78%) |  |
| Yes | 73 (21%) | 19 (22%) |  |
| **Alcohol history** |  |  | 0.2 |
| No | 277 (81%) | 63 (74%) |  |
| Yes | 65 (19%) | 22 (26%) |  |
| **Hypertension history** |  |  | >0.9 |
| No | 256 (75%) | 64 (75%) |  |
| Yes | 86 (25%) | 21 (25%) |  |
| **Diabetes history** |  |  | 0.5 |
| No | 301 (88%) | 77 (91%) |  |
| Yes | 41 (12%) | 8 (9.4%) |  |
| **Hyperlipidemia history** |  |  | 0.4 |
| No | 321 (94%) | 82 (96%) |  |
| Yes | 21 (6.1%) | 3 (3.5%) |  |
| **Coronary heart disease history** |  |  | 0.6 |
| No | 326 (95%) | 80 (94%) |  |
| Yes | 16 (4.7%) | 5 (5.9%) |  |
| **Stroke history** |  |  | 0.6 |
| No | 323 (94%) | 79 (93%) |  |
| Yes | 19 (5.6%) | 6 (7.1%) |  |
| **Prior VTE history** |  |  | 0.5 |
| No | 340 (99%) | 84 (99%) |  |
| Yes | 2 (0.6%) | 1 (1.2%) |  |
| **Preop anticoagulant use** |  |  | >0.9 |
| No | 323 (94%) | 80 (94%) |  |
| Yes | 19 (5.6%) | 5 (5.9%) |  |
| **Preop Caprini score (per point)** | 2 ± 1 | 2 ± 1 | 0.3 |
| **Preop D-dimer (mg/L)** | 0.63 ± 1.23 | 0.75 ± 1.76 | 0.9 |
| **Preop fibrinogen (g/L)** | 2.99 ± 0.80 | 3.05 ± 0.84 | 0.7 |
| **Preop FDP (ug/mL)** | 1.58 ± 2.36 | 1.78 ± 3.03 | 0.7 |
| **Preop PT (s)** | 12.96 ± 0.82 | 13.19 ± 0.83 | 0.021 |
| **Preop APTT (s)** | 33.9 ± 4.0 | 34.3 ± 3.4 | 0.4 |
| **Preop INR** | 0.98 ± 0.07 | 1.00 ± 0.07 | 0.020 |
| **Preop platelets (10^9/L)** | 218 ± 60 | 215 ± 58 | 0.9 |
| **Preop RBC (10^12/L)** | 4.54 ± 0.55 | 4.51 ± 0.52 | >0.9 |
| **Preop WBC (10^9/L)** | 7.17 ± 2.54 | 7.02 ± 2.32 | 0.6 |
| **Preop neutrophil (%)** | 66 ± 11 | 67 ± 10 | 0.2 |
| **Preop neutrophils abs (10^9/L)** | 4.86 ± 2.44 | 5.81 ± 9.49 | 0.9 |
| **Preop monocyte (%)** | 7.36 ± 2.64 | 7.13 ± 2.07 | 0.7 |
| **Preop lymphocyte (%)** | 25 ± 9 | 23 ± 9 | 0.2 |
| **Preop albumin (g/L)** | 42 ± 4 | 42 ± 4 | 0.4 |
| **Preop hemoglobin (g/L)** | 134 ± 17 | 134 ± 17 | 0.8 |
| **Preop total protein (g/L)** | 70 ± 6 | 71 ± 6 | 0.4 |
| **Preop creatinine (umol/L)** | 68 ± 16 | 62 ± 12 | 0.006 |
| **Preop urea (mmol/L)** | 5.62 ± 1.94 | 5.39 ± 1.73 | 0.3 |
| **Preop uric acid (umol/L)** | 298 ± 99 | 288 ± 88 | 0.3 |
| **Preop sodium (mmol/L)** | 140 ± 3 | 140 ± 3 | 0.6 |
| **Preop potassium (mmol/L)** | 3.90 ± 0.34 | 3.89 ± 0.34 | 0.8 |
| **Preop calcium (mmol/L)** | 2.26 ± 0.11 | 2.26 ± 0.13 | 0.5 |
| **Preop chloride (mmol/L)** | 105 ± 3 | 104 ± 4 | 0.6 |
| **Preop glucose (mmol/L)** | 6.10 ± 1.75 | 5.82 ± 1.43 | 0.049 |
| **Preop total cholesterol (mmol/L)** | 4.38 ± 0.91 | 4.27 ± 0.84 | 0.4 |
| **Preop triglyceride (mmol/L)** | 1.70 ± 2.20 | 1.41 ± 0.81 | 0.038 |
| **Preop HDL-C (mmol/L)** | 1.30 ± 0.36 | 1.34 ± 0.32 | 0.2 |
| **Preop LDL-C (mmol/L)** | 2.68 ± 0.75 | 2.59 ± 0.69 | 0.3 |
| **Preop total bilirubin (umol/L)** | 11.2 ± 5.1 | 13.9 ± 6.7 | <0.001 |
| **Preop direct bilirubin (umol/L)** | 3.24 ± 1.72 | 3.57 ± 2.12 | 0.3 |
| **Preop ALT (U/L)** | 26 ± 25 | 22 ± 14 | 0.043 |
| **Preop AST (U/L)** | 26 ± 32 | 27 ± 23 | 0.7 |
| **Tumor location** |  |  | 0.3 |
| Deep/Other | 84 (25%) | 27 (32%) |  |
| Frontal | 112 (33%) | 30 (35%) |  |
| Non-frontal lobar | 109 (32%) | 19 (22%) |  |
| Ventricle | 37 (11%) | 9 (11%) |  |
| **Tumor laterality** |  |  | 0.5 |
| Bilateral | 52 (15%) | 17 (20%) |  |
| Left | 138 (40%) | 31 (36%) |  |
| Right | 152 (44%) | 37 (44%) |  |
| **Tumor spread** |  |  | 0.3 |
| Localized | 108 (32%) | 22 (26%) |  |
| Regional | 234 (68%) | 63 (74%) |  |
| **Tumor max diameter (cm)** | 4.81 ± 1.57 | 5.05 ± 1.74 | 0.2 |
| **Recurrent glioma** |  |  | 0.6 |
| No | 299 (87%) | 76 (89%) |  |
| Yes | 43 (13%) | 9 (11%) |  |
| ^1^n (%); Mean ± SD | | | |
| ^2^Pearson's Chi-squared test; Wilcoxon rank sum test; Fisher's exact test | | | |

**Table S1. Baseline characteristics of the study cohort for VTE prediction, stratified by training and test sets.** Clinically significant VTE is included for descriptive purposes only to show its distribution across training and test sets, and was not used as a predictor or outcome in model training. This table summarizes the demographic characteristics, medical history, tumor-related variables, preoperative (Preop) risk assessment variables, and laboratory measurements considered in the development and evaluation of the VTE prediction models. Only variables available before surgery were included. Abbreviations: VTE, venous thromboembolism; DVT, deep vein thrombosis; PE, pulmonary embolism; BMI, body mass index; Preop, preoperative; FDP, fibrin degradation products; PT, prothrombin time; APTT, activated partial thromboplastin time; INR, international normalized ratio; RBC, red blood cell count; WBC, white blood cell count; HDL-C, high-density lipoprotein cholesterol; LDL-C, low-density lipoprotein cholesterol; ALT, alanine aminotransferase; AST, aspartate aminotransferase; SD, standard deviation.
