# Supplementary material for: GLOBE: an explainable machine learning platform for preoperative prediction of thromboembolism and neurological deterioration in patients with glioma
Source: Front Neurosci. 2026 Jun 1;20:1801112. doi: 10.3389/fnins.2026.1801112 (PMC13265528; doi:10.3389/fnins.2026.1801112)
Supplement: Supplementary file 2 [file Table_2.docx]

|  | **Train**  N = 342^1^ | **Test**  N = 85^1^ | **p-value**^2^ |
| --- | --- | --- | --- |
| **PND** |  |  | >0.9 |
| No | 221 (65%) | 55 (65%) |  |
| Yes | 121 (35%) | 30 (35%) |  |
| **Age (per year)** | 53 ± 14 | 53 ± 15 | 0.8 |
| **Sex** |  |  | 0.7 |
| Female | 165 (48%) | 43 (51%) |  |
| Male | 177 (52%) | 42 (49%) |  |
| **BMI (kg/m^2)** | 23.48 ± 2.89 | 23.59 ± 2.66 | 0.6 |
| **Smoking history** |  |  | 0.5 |
| No | 266 (78%) | 69 (81%) |  |
| Yes | 76 (22%) | 16 (19%) |  |
| **Alcohol history** |  |  | 0.6 |
| No | 274 (80%) | 66 (78%) |  |
| Yes | 68 (20%) | 19 (22%) |  |
| **Hypertension history** |  |  | 0.8 |
| No | 257 (75%) | 63 (74%) |  |
| Yes | 85 (25%) | 22 (26%) |  |
| **Diabetes history** |  |  | 0.4 |
| No | 305 (89%) | 73 (86%) |  |
| Yes | 37 (11%) | 12 (14%) |  |
| **Hyperlipidemia history** |  |  | 0.4 |
| No | 321 (94%) | 82 (96%) |  |
| Yes | 21 (6.1%) | 3 (3.5%) |  |
| **Coronary heart disease history** |  |  | 0.4 |
| No | 327 (96%) | 79 (93%) |  |
| Yes | 15 (4.4%) | 6 (7.1%) |  |
| **Stroke history** |  |  | 0.4 |
| No | 320 (94%) | 82 (96%) |  |
| Yes | 22 (6.4%) | 3 (3.5%) |  |
| **Prior VTE history** |  |  | 0.5 |
| No | 340 (99%) | 84 (99%) |  |
| Yes | 2 (0.6%) | 1 (1.2%) |  |
| **Preop anticoagulant use** |  |  | 0.6 |
| No | 324 (95%) | 79 (93%) |  |
| Yes | 18 (5.3%) | 6 (7.1%) |  |
| **Preop Caprini score (per point)** | 2 ± 1 | 2 ± 1 | 0.081 |
| **Preop D-dimer (mg/L)** | 0.63 ± 1.23 | 0.76 ± 1.77 | 0.5 |
| **Preop fibrinogen (g/L)** | 2.99 ± 0.80 | 3.05 ± 0.84 | 0.4 |
| **Preop FDP (ug/mL)** | 1.59 ± 2.43 | 1.73 ± 2.80 | 0.5 |
| **Preop PT (s)** | 12.98 ± 0.82 | 13.09 ± 0.86 | 0.4 |
| **Preop APTT (s)** | 33.9 ± 3.9 | 34.6 ± 3.8 | 0.3 |
| **Preop INR** | 0.99 ± 0.07 | 0.99 ± 0.08 | 0.5 |
| **Preop platelets (10^9/L)** | 219 ± 60 | 209 ± 60 | 0.10 |
| **Preop RBC (10^12/L)** | 4.55 ± 0.54 | 4.47 ± 0.57 | 0.3 |
| **Preop WBC (10^9/L)** | 7.21 ± 2.59 | 6.88 ± 2.05 | 0.5 |
| **Preop neutrophil (%)** | 66 ± 11 | 67 ± 11 | 0.3 |
| **Preop neutrophils abs (10^9/L)** | 5.12 ± 5.22 | 4.76 ± 2.01 | 0.9 |
| **Preop monocyte (%)** | 7.31 ± 2.56 | 7.31 ± 2.45 | 0.7 |
| **Preop lymphocyte (%)** | 25 ± 9 | 23 ± 9 | 0.11 |
| **Preop albumin (g/L)** | 42 ± 4 | 41 ± 4 | >0.9 |
| **Preop hemoglobin (g/L)** | 135 ± 17 | 133 ± 18 | 0.4 |
| **Preop total protein (g/L)** | 70 ± 6 | 70 ± 6 | 0.9 |
| **Preop creatinine (umol/L)** | 67 ± 16 | 65 ± 15 | 0.4 |
| **Preop urea (mmol/L)** | 5.58 ± 1.89 | 5.57 ± 1.97 | 0.9 |
| **Preop uric acid (umol/L)** | 296 ± 97 | 296 ± 95 | 0.7 |
| **Preop sodium (mmol/L)** | 140 ± 3 | 140 ± 3 | 0.6 |
| **Preop potassium (mmol/L)** | 3.90 ± 0.34 | 3.89 ± 0.34 | 0.5 |
| **Preop calcium (mmol/L)** | 2.26 ± 0.12 | 2.25 ± 0.11 | 0.8 |
| **Preop chloride (mmol/L)** | 105 ± 3 | 104 ± 4 | 0.5 |
| **Preop glucose (mmol/L)** | 5.98 ± 1.67 | 6.32 ± 1.78 | 0.045 |
| **Preop total cholesterol (mmol/L)** | 4.38 ± 0.89 | 4.26 ± 0.92 | 0.3 |
| **Preop triglyceride (mmol/L)** | 1.63 ± 2.06 | 1.70 ± 1.75 | 0.6 |
| **Preop HDL-C (mmol/L)** | 1.32 ± 0.36 | 1.25 ± 0.35 | 0.086 |
| **Preop LDL-C (mmol/L)** | 2.68 ± 0.74 | 2.58 ± 0.73 | 0.3 |
| **Preop total bilirubin (umol/L)** | 11.6 ± 5.3 | 12.5 ± 6.6 | 0.4 |
| **Preop direct bilirubin (umol/L)** | 3.30 ± 1.72 | 3.31 ± 2.15 | 0.5 |
| **Preop ALT (U/L)** | 25 ± 25 | 25 ± 16 | 0.4 |
| **Preop AST (U/L)** | 26 ± 33 | 27 ± 20 | 0.6 |
| **Tumor location** |  |  | 0.2 |
| Deep/Other | 87 (25%) | 24 (28%) |  |
| Frontal | 107 (31%) | 35 (41%) |  |
| Non-frontal lobar | 108 (32%) | 20 (24%) |  |
| Ventricle | 40 (12%) | 6 (7.1%) |  |
| **Tumor laterality** |  |  | 0.7 |
| Bilateral | 53 (15%) | 16 (19%) |  |
| Left | 137 (40%) | 32 (38%) |  |
| Right | 152 (44%) | 37 (44%) |  |
| **Tumor spread** |  |  | 0.8 |
| Localized | 103 (30%) | 27 (32%) |  |
| Regional | 239 (70%) | 58 (68%) |  |
| **Tumor max diameter (cm)** | 4.81 ± 1.57 | 5.04 ± 1.76 | 0.2 |
| **Recurrent glioma** |  |  | 0.2 |
| No | 304 (89%) | 71 (84%) |  |
| Yes | 38 (11%) | 14 (16%) |  |
| ^1^n (%); Mean ± SD | | | |
| ^2^Pearson's Chi-squared test; Wilcoxon rank sum test; Fisher's exact test | | | |

**Table S2. Baseline characteristics of the study cohort for PND prediction, stratified by training and test sets.** This table summarizes the demographic characteristics, medical history, tumor-related variables, preoperative (Preop) risk assessment variables, and laboratory measurements considered in the development and evaluation of the PND prediction models. Only variables available before surgery were included. Abbreviations: BMI, body mass index; Preop, preoperative; FDP, fibrin degradation products; PT, prothrombin time; APTT, activated partial thromboplastin time; INR, international normalized ratio; RBC, red blood cell count; WBC, white blood cell count; HDL-C, high-density lipoprotein cholesterol; LDL-C, low-density lipoprotein cholesterol; ALT, alanine aminotransferase; AST, aspartate aminotransferase; SD, standard deviation.
